# The Neurophysiologist Perspective into MS Plasticity

**DOI:** 10.3389/fneur.2015.00193

**Published:** 2015-09-03

**Authors:** Elise Houdayer, Giancarlo Comi, Letizia Leocani

**Affiliations:** ^1^Experimental Neurophysiology Unit, Institute of Experimental Neurology (INSPE), San Raffaele Scientific Institute, Milan, Italy; ^2^University Vita-Salute San Raffaele, San Raffaele Scientific Institute, Milan, Italy

**Keywords:** multiple sclerosis, transcranial magnetic stimulation, non-invasive brain stimulation, electroencephalography, plasticity

## Abstract

Multiple sclerosis (MS) is a frequent, highly debilitating inflammatory demyelinating disease, starting to manifest in early adulthood and presenting a wide variety of symptoms, which are often resistant to pharmacological treatments. Cortical dysfunctions have been demonstrated to be key components of MS condition, and plasticity of the corticospinal motor system is highly involved in major MS symptoms, such as fatigue, spasticity, or pain. Cortical dysfunction in MS can be studied with neurophysiological tools, such as electroencephalography (EEG) and related techniques (evoked potentials) or transcranial magnetic stimulation (TMS). These techniques are now widely used to provide essential elements of MS diagnosis and can also be used to modulate plasticity. Indeed, the recent development of non-invasive brain stimulation techniques able to induce cortical plasticity, such as repetitive TMS or transcranial direct current stimulation, has brought promising results as add-on treatments. In this review, we will focus on the use of these tools (EEG and TMS) to study plasticity in MS and on the major techniques used to modulate plasticity in MS.

## Introduction

Multiple sclerosis (MS) is usually described as an inflammatory demyelinating disease involving mainly the white matter. However, axonal loss (1) and cortical damage (2–6) are also important clinical features of the disease. The first demonstration of cortical involvement was reported in the 1980s, showing losses of orientation-specific contrast sensitivity and abnormal visual evoked potentials (VEPs) in MS (7, 8). The role of cortical damage in the disease course and clinical deficits has been since then further investigated and plasticity of the corticospinal motor system has been identified as a key component of major debilitating symptoms, such as fatiguability or spasticity (9–19).

Neurophysiological examinations are thus of primary importance in the clinical care of MS. They allow both the investigation of corticospinal sensorimotor mechanisms involved in the disease, using electroencephalography (EEG), electromyography (EMG), and transcranial magnetic stimulation (TMS), and also allow clinicians to directly act on deficient cortical circuits to improve subjects’ condition, using non-invasive brain stimulation (NIBS), such as repetitive TMS (rTMS), theta burst stimulation, transcranial direct current stimulation (tDCS), or using peripheral nerve stimulation, such as transcutaneous electrical nerve stimulation (TENS).

In this review, we will focus on the importance of neurophysiological tools to study and modulate plasticity in MS to help treat major symptoms of the disease.

## Exploring Cortical Plasticity in MS

### Event-related EEG oscillations

EEG represents an important exploratory tool in clinical neurophysiology practice in general and in particular in the care of MS, especially using multimodal evoked potentials (EPs), such as somatosensory evoked potentials (SEPs), auditory evoked potentials (AEPs), or VEPs. These measurements allow indeed a quantitative assessment of the system function targeted by the examination.

Apart from the evoked activity, cortico-thalamo-cortical loops can be studied using induced EEG activity in relation to internal or external events. In particular, event-related desynchronization/synchronization (ERD/ERS) of the sensorimotor mu (8–12 Hz) and beta (13–25 Hz) rhythms is strongly related to the cortical motor control (20–22). ERD represents an attenuation of the EEG signal amplitude. Mu and beta ERD, predominating over the sensorimotor cortical areas contralateral to movement, initiate about 1.5 s before movement onset and are maximal at movement onset. Mu/beta ERD, usually observed before and during self-paced voluntary movements (23, 24), reaction time paradigms (25), passive movements (26, 27), or motor imagery (28), reflect the activation of cortical motor/premotor areas involved in motor planning. Beta ERS corresponds to a brisk, intense amplitude increase following movement termination, observed in the beta band. Beta ERS would be related to a post-event inhibitory period strongly related to sensory reafferentation (29–32). The mu/beta ERD/ERS analysis is thus a robust method to study the cortical processing of motor control.

Beta ERD was abnormally increased in the fronto-central regions in fatigued MS subjects, compared to non-fatigued subjects or controls (11). The study involved non-disabled subjects [with score ≤1.5 according to the Expanded Disability Status Scale (EDSS)]. Non-fatigued subjects did not show abnormal mu/beta ERD/ERS. Conversely, beta ERS was significantly lower in fatigued MS participants over fronto-central areas. These abnormal ERD/ERS patterns were significantly correlated with the amount of fatigue. Such increased beta ERD and decreased beta ERS reflected an increased cortico-thalamo-cortical activity in fatigued MS subjects, consistent with the central origin of fatigue in MS, and suggesting an over-activity of frontal structures (probably the supplementary motor area). In another study involving more severe MS participants, the authors showed a significant correlation between mu ERD onset and T1/T2 total lesion volume, the more severe subjects having higher lesion loads and more delayed mu ERD (33). These results imply that, with the progression of the disease, the extent of brain lesion load affects cortico-cortical and cortico-subcortical activity related to motor planning.

### Long-latency reflexes

Long-latency reflexes (LLRs) are muscular responses elicited by electrical stimulations of mixed nerves during slight contraction of the targeted muscle. In particular, LLR-II would be the most reliable (34) and would represent a transcortical reflex (35–40). LLR-II represents an important neurophysiological tool to study simultaneously the sensory-motor corticospinal tracts and intracortical circuits. There is a strong correlation between LLR-II latencies and the sum of latencies of the N20 SEP and motor-evoked potential (MEP) evoked by TMS, suggesting that the three phenomena (LLR-II, SEP, and MEP) are essentially conducted along the same fibers (39). The cortical relay time (CRT) can be obtained by subtracting the sum of the latencies of N20 and of MEP to the LLR-II latency. CRT is usually consistent with polysynaptic or oligosynaptic intracortical transmission (41). Delayed or absent LLRs in MS were revealed in the early 90s, demonstrating the relevance of studying simultaneously LLRs and SEPs to evaluate afferent and efferent pathways in MS (42, 43). More specifically, the CRT was reported prolonged in people with definite MS (44, 45). Tataroglu and colleagues demonstrated also prolonged LLR-II, N20 SEP, and MEP latencies in MS. The CRT was not correlated with the clinical form of the disease or with its duration, in contrast to the other measurements. Bonfiglio and colleagues showed only weak differences between people with MS and controls in terms of afferent (N20) or efferent (MEP) conduction times, but demonstrated strong differences of LLR latencies between both groups (45). Moreover, CRT was greatly prolonged in MS compared to controls, and not only in subjects who had severe slowing of central sensory and/or motor conduction. The CRT increase did not correlate with disease duration. This study showed how slowing of intracortical sensorimotor circuits greatly contributes to the delayed LLR-II latencies in MS. LLR recording may thus be useful to detect dysfunctions of the intracortical sensorimotor pathway in MS. Attention can be directed on the fact that these intracortical sensorimotor disorders are present in most of MS subjects, independently from the disease duration and even in non-severe forms.

### Transcranial magnetic stimulation

TMS was initially used in MS to measure central motor conduction time (CMCT) to evaluate the effects of demyelination on neuronal conduction. CMCT is indeed significantly prolonged in MS (46–48). Moreover, depending on the paradigm used, single or paired-pulse TMS allows the investigation of the whole corticospinal tract integrity, including intracortical excitability. Such paradigms have been used in MS and showed increased resting motor threshold (RMT), or absent MEPs in most of subjects, demonstrating abnormal excitability of pyramidal neuron membrane (46, 47, 49). Increased threshold and reduced cortical silent period (CSP, a measure of intracortical GABAb transmission) were demonstrated characteristic of “relapsing” subjects. These participants also lacked short-interval intracortical inhibition (SICI), a measurement of intracortical GABA-a interneuronal transmission (50–52). Normal threshold and prolonged CSP were observed in the “remitting” phase (53). Strong correlations were shown between hand motor function (measured with the Purdue Pegboard score) and RMT, MEP amplitude/latencies in relapsing-remitting MS (54). Relapsing-remitting subjects had lower RMT and higher MEP amplitudes than subjects with secondary progressive MS, who had significantly higher RMT and smaller MEPs than controls (48, 55). Secondary progressive MS also showed lower amounts of SICI than relapsing-remitting form and than healthy controls, directly demonstrating an alteration of the intracortical GABAergic transmission in MS (55). These TMS measures correlated with EDSS scores, revealing normal TMS measures in subjects with lower EDSS scores and abnormal corticospinal excitability in people with higher EDSS scores (i.e., higher disability), demonstrating that TMS evaluation is of importance in quantifying MS disease severity (48, 55, 56). Also, changes in the balance of intracortical excitation and inhibition, favoring excitation, have been reported using paired-pulse TMS after high-dose corticosteroids in relation to a relapse (57). More studies are needed in order to ascertain the respective role of lesion location (e.g., motor or extra-motor relapse), of spontaneous recovery and of treatment administration.

TMS can also be used as a non-invasive tool able to interfere temporarily with a specific cortical activity in order to investigate its particular role. To this aim, single pulse TMS has been used to investigate the role of ipsilateral motor/premotor cortex hyperactivity during a simple reaction time task in MS (58). The authors applied a suprathreshold TMS pulse targeting, in different sessions, the contralateral and ipsilateral hand motor cortices or the ipsilateral dorsal premotor cortex during a simple reaction time task. They showed that the concomitant stimulation of the contralateral primary motor cortex increased significantly the reaction times in both people with MS and controls. Conversely, stimulation of the ipsilateral motor/premotor cortex increased reaction times only in MS, and not in controls. These changes in reaction times, however, did not correlate with hand motor function tests or with the total brain lesion load. The authors concluded thus that the ipsilateral hyperactivity might be a “functionally relevant, yet limited adaptive response to chronic brain injury in MS patients.”

## Modulating Cortical Plasticity in MS

### Non-invasive brain stimulation

Non-invasive brain stimulation techniques are relatively new tools for modulating cortical excitability to provide symptomatic treatments in a large range of neurologic and psychiatric diseases. Among them, rTMS and tDCS have been widely studied and proven effective in conditions, such as Parkinson’s disease, stroke, or dystonia (59–62). Since these techniques have been particularly applied in the field of neurorehabilitation, a special interest rose to improve specific dysfunctions of subjects with MS.

Cortical plasticity can indeed by induced in MS. Subjects with moderately severe stable MS showed the same rapid-onset motor plasticity than healthy subjects, despite motor impairment and central nervous system injuries (63). The authors used paired-associative stimulation (PAS), a NIBS protocol modeling long-term synaptic potentiation (LTP) (64), combining repetitive electric nerve stimulation with TMS of the contralateral motor cortex. In both groups (MS and controls), PAS induced an increase in corticospinal excitability and improved motor learning performances equally in subjects with MS and controls. On the other hand, PAS-induced plasticity was reduced in relapsing-remitting MS subjects suffering incomplete or absent recovery (65). The authors showed that PAS-induced plasticity (measured with MEPs and SEPs amplitude and latencies) and age could contribute to predict symptom recovery after a relapse.

One of the first applications of NIBS in MS has been to reduce spasticity. Indeed, rTMS is able to modulate the presynaptic inhibition of the soleus Ia afferents mediating the stretch reflex (66, 67). Centonze and colleagues first applied low (inhibitory) and high (excitatory) frequency rTMS over the leg primary motor cortex in 19 subjects with remitting MS and showed that a single session of high-frequency rTMS (5 Hz) could reduce the amplitude of the H/M ratio of the soleus H reflex and increase MEP amplitude (18). Two consecutive weeks of 5 Hz rTMS treatment decreased H/M amplitude ratio as well as spasticity [directly measured on Modified Ashworth Scale (MAS) mean score], up to 1 week after the end of treatment (18). In a pilot study using the H-coil, which is able to deliver a wider and deeper magnetic field than the regular focal coils without the need to increase the stimulation intensity (68), 3 weeks of treatment with 20 Hz rTMS over the leg area of subjects with progressive MS could improve walking and reduce spasticity more than rehabilitation alone (69). Intermittent theta burst stimulation (iTBS), which represents another way of using high-frequency rTMS to increase corticospinal excitability (70), has also been reported to reduce spasticity (MAS scores and H/M amplitude ratio) in the remitting phase of MS for up to 2 weeks after the end of the 2-week stimulation protocol (71, 72). The effects of iTBS, combined with exercise therapy, were potentiated with respect to the two treatments alone, suggesting the association of these two rehabilitation methods as a promising strategy (72). Conversely, iTBS-induced LTP was reported absent in subjects with primary progressive MS, who also presented lesser amounts of platelet-derived growth factor (73), a molecule considered neuroprotective (74) and favoring LTP (75).

NIBS techniques have also been used to treat fatigue in MS. Indeed, cortical involvement in fatigue mechanisms was demonstrated through impaired intracortical inhibition (13), dysfunction of inhibitory mechanisms engaged after movement termination (11, 76), in line with neuroimaging evidence (6). Positron emission tomography at rest revealed metabolic abnormalities of frontal cortex and basal ganglia (77) and functional magnetic resonance imaging during motor activity showed dysfunction of cortical and subcortical areas involved in motor planning (12). tDCS, another NIBS method for inducing long-term modulation of cortical excitability (78, 79), has been recently explored to reduce fatigue in MS. Anodal (excitatory) tDCS of the motor cortex applied for 5 days in 25 MS subjects (22 relapsing-remitting) could improve fatigue impact scale (FIS) scores by about 30% in 65% of participants (80). These benefits were still present 3 weeks after the end of treatment. More recently, 5 days of bilateral anodal tDCS over the primary somatosensory cortical areas were able to decrease fatigue (modified FIS scores) in 10 MS subjects (81). Anodal tDCS over the somatosensory cortex could also reduce tactile sensory deficits by improving discriminatory thresholds at the grating orientation task and increasing the visual analog scale (VAS) for sensory scores in 20 remitting subjects (82).

Another application of NIBS in MS has been neuropathic pain. Central neuropathic pain is influenced by functional changes at the supra-spinal level, in various components involved in pain perception. In particular, the thalamic nuclei, limbic system, sensorimotor, and insular cortices function in a hyperactivated state. A lack of intracortical inhibition would also be involved in central neuropathic pain (83). Based on these observations, epidural and transcranial stimulation of the motor cortex, modulating pain perception through indirect neural networks, have been applied in humans for the treatment of drug-resistant neuropathic pain (84). Five days of anodal tDCS over the primary motor cortex reduced pain (assessed by VAS for pain and McGill questionnaire) and improved quality of life in 19 remitting MS subjects (85), up to 3 weeks after the end of treatment.

These studies demonstrated that neuromodulation of cortical plasticity using NIBS can have diverse applications to benefit people with MS. NIBS over M1 might reduce spasticity and neuropathic pain through an increase in corticospinal excitability (18, 70, 86), while the positive effects on fatigue might depend on cortico-cortical and/or cortico-subcortical mechanisms (80, 81).

### Transcutaneous electrical nerve stimulation

Transcutaneous electrical nerve stimulation is used in the treatment of acute or chronic pain symptoms (87). TENS usually consists on the use of small battery-powered devices delivering alternative current through cutaneous electrodes placed near the painful area. TENS efficacy depends on the intensity and frequency of stimulation. TENS activates large diameter afferent fibers, which in the central nervous system may activate descending inhibitory circuits reducing hyperalgesia (88, 89). In animal models, low and high-frequency TENS reduce dorsal horn neuronal activity (90–93). High-frequency TENS also reduces central neuronal sensitization and release of glutamate and substance P in the spinal chord dorsal horn in preclinical models of inflammation (94, 95). In MS, TENS has been reported to reduce spasticity, pain, and muscle spasms (96, 97). Recently, a TMS study investigated the effects of a 3-week TENS treatment on cortical map representation (98). TENS, applied on the median nerve region (thenar eminence) of the most impaired hand 1 h a day for 3 weeks, was associated with decreased cortical map area of hand muscle representation, without modifying RMT or MEP amplitude. These findings were interpreted as reflecting reorganization in the cortical motor representation rather than a temporary decline in corticospinal excitability, suggesting that TENS can induce cortical plastic changes in MS.

## Conclusion

A variety of neurophysiology tools can significantly help in the investigation and reinforcement of neuroplasticity in MS. Importantly, the development of NIBS techniques is bringing new possibilities for add-on treatment strategies. Thus, the combination of these tools could help personalize treatments for people with MS.

## Conflict of Interest Statement

The authors declare that the research was conducted in the absence of any commercial or financial relationships that could be construed as a potential conflict of interest.
